# SIRT1 retention in elongating spermatids interferes with histone displacement by counteracting MOF-dependent H4K16 acetylation

**DOI:** 10.3389/fcell.2025.1524919

**Published:** 2025-08-29

**Authors:** Veronica Porreca, Teresa Chioccarelli, Francesco Albano, Valeria Nittoli, Giulia Ricci, Concetta Ambrosino, Rosanna Chianese, Vincenza Grazia Mele, Antonella Migliaccio, Mariano Stornaiuolo, Antonio Suglia, Gilda Cobellis, Francesco Manfrevola

**Affiliations:** ^1^ Department of Experimental Medicine, University of Campania “Luigi Vanvitelli”, Naples, Italy; ^2^ Department of Biology, University of Naples Federico II, Naples, Italy; ^3^ Biogem, Biology and Molecular Genetics Institute, Ariano Irpino, Italy; ^4^ Institute of Experimental Endocrinology and Oncology (IEOS), CNR, Naples, Italy; ^5^ Department of Pharmacy, University of Naples Federico II, Naples, Italy; ^6^ University Rennes, Inserm, EHESP, Irset (Institut de Rechercheen Aanté, environnement et travail) – UMR_S 1085, Rennes, France

**Keywords:** histone displacement, SIRT1, MOF, histone post-translational modification, chromatin remodeling, histone acetylation, spermiogenesis, cannabinoid receptor

## Abstract

**Introduction:**

The histone H4 hyperacetylation (i.e., acetylation of H4 at lysines -K5, -K8, -K12, and -K16, here reported as H4tetraAc) occurs in elongating spermatids (eSPTs) during spermiogenesis. Although it is critically involved in histone displacement, the mechanistic involvement of histone -acetyltransferases (HATs) and -deacetylases (HDACs) in the pathway underlying H4 hyperacetylation is poorly defined. Here, we investigate the involvement of SIRT1 deacetylase, and its functional interaction with the histone acetyltransferase MOF, in regulating H4 hyperacetylation underlying histone-to-protamine exchange.

**Methods:**

Exploiting the cannabinoid receptor 1 (*Cb1*) null mice (Cb1^−/−^) as a model of impaired histone displacement, we assessed in eSPTs the expression and the localization of SIRT1 in combination with the enrichment of H4tetraAc and the relative monoacetylated forms (H4-K5ac, -K8ac, -K12ac and -K16ac), by Western Blot and immunohistochemistry analyses. Then, focusing on SIRT1 interaction with MOF HAT by protein immunoprecipitation experiments, we verified the H4K16ac and H4TetraAc enrichment in eSPTs in response to *ex vivo* SIRT1 inhibition by using the selective EX-527 inhibitor.

**Results:**

We show that the hyperacetylation of histone H4 occurs progressively in steps 8–9 eSPTs and bursts in step 10 eSPTs, appearing inversely correlated to the expression pattern of SIRT1, being SIRT1 present in step 8, detectable in step 9 and absent in step 10 eSPTs. The abnormal SIRT1 retention in step 10 eSPTs of Cb1^−/−^ mice, despite the observed enrichment of H4-K5ac, -K8ac, and -K12ac, counteracts the H4 hyperacetylation burst by limiting the H4 acetylation at lysine K16. Mechanistically, SIRT1 directly or indirectly interacts with and negatively regulates MOF acetyltransferase, specifically affecting its acetylation status and protein content, thereby interfering with H4K16 acetylation. Counteracting the MOF/SIRT1 interaction by SIRT1 inhibition in *ex vivo* Cb1^−/−^ testis, both MOF protein content and acetylation status increase, downstream promoting recovery of H4K16ac and H4tetraAc in step 10 eSPT, and full rescue of histone displacement.

**Conclusion:**

These results underscore the key involvement of SIRT1–MOF axis in modulating H4K16 acetylation. Our findings provide mechanistic insights into H4K16 acetylation pathway in eSPTs and support the key role of H4K16ac in chromatin remodeling underlying histone displacement.

## Introduction

During the morphogenesis of round spermatids (SPTs) into spermatozoa (SPZs) (i.e., spermiogenesis), a dramatic chromatin remodeling molecularly orchestrated by genome-wide histone displacement and protamine incorporation occurs (Cacciola et al., 2013a; Awe and Renkawitz-Pohl, 2010). Although most histones are replaced by protamines, a small fraction of the core-histone chromatin (1%–5% in mice and 10%–15% in humans) is physiologically retained in mature SPZs with potential functional implications. Indeed, in both mice and humans, nucleosomes are not randomly distributed in the sperm chromatin and appear labeled by various histone post-translational modifications (HPTMs) (Hammoud et al., 2009; Luense et al., 2016; Jung et al., 2017; Yoshida et al., 2018; Yamaguchi et al., 2018; Martinez et al., 2023). These sperm epigenetic markers promote a more relaxed and transcriptionally flexible chromatin structure, conceived as a key regulator of embryonic development, potentially responsible for paternal epigenetic inheritance (Brykczynska et al., 2010; van der Heijden et al., 2006; Erkek et al., 2013; Jung et al., 2017; Torres-Flores and Hernández-Hernández, 2020). In mouse, SPT differentiation can be morphologically divided into 16 developmental steps (including round, elongating, condensing, and condensed SPTs), where each step is associated with molecular and morphological events.

The H4-hyperacetylated histone marker is a result of acetylation of the N-terminal tail of histone H4 at the lysine residues -K5, -K8, -K12, and -K16 (reported herein as H4 hyperacetylation or H4tetraAc) and is observed in elongating SPTs (eSPTs) from steps 8 to 11 (Chioccarelli et al., 2020a; Hazzouri et al., 2000). It acts as a prerequisite for histone displacement as any interference with H4 hyperacetylation leads to inefficient histone removal (Bell et al., 2014). This massive wave of H4 hyperacetylation makes chromatin prone to the repair of transient DNA strand breaks as well as removal and degradation of acetylated histones. The latter are mediated by testis-specific bromodomain protein (BRDT) and spermatoproteasome (PA200-20S/α4s), respectively (Qian et al., 2013; Rathke et al., 2014; Hoghoughi et al., 2017; Hazzouri et al., 2000; Bao and Bedford, 2016; Moritz and Hammoud, 2022). In particular, the H4K5ac and H4K8ac histone markers are known to act as binding sites for BRDT. This chromatin reader causes displacement of acetylated histones through the interaction with SMARCE1 a member of the SWI/SNF family chromatin remodeling complex with histone eviction activity (Chioccarelli et al., 2020a).

Although the occurrence of H4 hyperacetylation is well established (Govin et al., 2007; Hazzouri et al., 2000), the involvement of selective histone acetyl transferases (HATs) modulating histone removal, such as TIP60 and CBP/p300 complexes (Doyon et al., 2004; Dong et al., 2017; Shiota et al., 2018), GCN5, and MOF (i.e., male on the first, also known as MYST1 or KAT8) (Lu et al., 2011; Luense et al., 2019), has emerged in recent times. In particular, the CBP/p300 HAT complex enhances acetylation of histone H4 on K5 and K8 residues that are useful for BRDT recruitment and activity (Shiota et al., 2018); on the other hand, ubiquitination of histone H2A and H2B induces transhistone acetylation of H4 at lysine K16 by enhancing MOF recruitment on the chromatin (Lu et al., 2010; Sin et al., 2012).

In this context, the involvement of NAD^+^-dependent histone deacetylases (Class III HDACs) in modulating histone displacement mechanism have been highlighted. Indeed SIRT1, a sirtuin family member belonging to the Class I sirtuin-subfamily, plays a key role in histone-to-protamine exchange. SIRT1 is expressed in several germ cell types, and the phenotype of germ cell-specific SIRT1 knockout male mice reveals that its deletion impairs histone removal by negatively affecting the acetylation of H4 at K5, K8, and K12 but not at K16, thus compromising H4tetraAc enrichment in eSPTs (Bell et al., 2014). In addition to typical histone substrates like H1K26ac, H3K9ac, and H4K16ac (Vaquero et al., 2004), SIRT1 targets several acetyltransferases, including Tip60, CBP, p300, MOF, GCN5, PCAF, and TAF1 (Chen et al., 2012). Interestingly, the activities of some HATs are regulated by SIRT1-mediated deacetylation (Chen et al., 2012; Pediconi et al., 2009; Bouras et al., 2005; Peng et al., 2012), indicating that histone deacetylases (HDACs) and HATs can cooperate in an antagonistic or unique manner with each other to establish histone acetylation levels in the same chromatin regions (Peserico and Simone, 2011; Peng et al., 2012). Such similar antagonistic or coordinated regulation between SIRT1 and the HATs modulating H4-hyperacetylation-based histone removal has not been assessed previously. In mouse testis, MOF is highly enriched in eSPTs, and its expression pattern is correlated with the progressive increase of H4K16ac (Lu et al., 2010). In addition, SIRT1 can deacetylate hMOF and regulate its HAT activity in human cells (Peng et al., 2012). Despite these findings, the potential SIRT1-mediated regulation of MOF activity as in H4K16 HAT in eSPTs represents a still unexplored aspect.

To investigate this topic, we used the cannabinoid receptor type 1 (CB1; alternatively indicated as CNR1) knockout (Cb1^−/−^) mice as a model of impaired histone displacement (Cacciola et al., 2013b) to characterize the involvement of SIRT1 in the regulation of the MOF-dependent burst of H4 hyperacetylation. The CB1 receptor is expressed in germ cells, especially in eSPTs (Cobellis et al., 2006), where it is implicated in histone displacement (Cacciola et al., 2013b). At the testis level, the *Cb1* gene deletion (Cb1^−/−^) interferes with the hyperacetylation of histone H4 and the efficiency of histone removal. Consequently, Cb1^−/−^ mice produce SPZs characterized by abnormal sperm histone content and poor chromatin quality parameters (i.e., damaged DNA, immature chromatin packaging, and elongated nuclear size) (Cacciola et al., 2013c; Chioccarelli et al., 2010; Chioccarelli et al., 2020b; Cacciola et al., 2013a). Aside from the impoverishment of H4tetraAc content, the expressions of other players involved in histone removal, such as *Cdyl* and *Brdt*, remain unaffected in the Cb1^−/−^ testis (Chioccarelli et al., 2020b). Moreover, the responsiveness of SIRT1 expression to CB1 activity has been demonstrated in several cell types (Zhang et al., 2018; Azar et al., 2020), thus highlighting the usefulness of the Cb1^−/−^ experimental model for understanding the role of SIRT1 in the acetylation pathways involved in histone removal.

In the present study, by systematically comparing testes from wild type (WT) and Cb1-null mice under both heterozygous (Cb1^+/−^) and homozygous (Cb1^−/−^) conditions; we analyzed SIRT1 expression in combination with the levels of H4tetraAc and its relative monoacetylated forms. Next, by focusing on the MOF/SIRT1 interaction, we characterized the SIRT1-dependent modulation of H4K16 acetylation in eSPTs by inhibiting its activity in *ex vivo* Cb1^−/−^ testis cultures or downregulating its level in estrogen-treated Cb1^−/−^ male mice, here properly used as histone displacement recovery model.

## Materials and methods

### Experimental animals

CD1-WT male mice or those carrying a *Cb1*-null mutation under heterozygous (Cb1^+/−^) or homozygous (Cb1^−/−^) conditions were used in this study (Ledent et al., 1999). The heterozygous mice (both male and female) were bred on a CD1 background (Charles River Laboratory, Lecco, Italy) and crossed to generate WT Cb1^+/−^ and Cb1^−/−^ male mice. All the animals were housed in a room with strictly controlled temperature (22°C ± 2°C), ventilation, and lighting (12-h light/dark cycles) and were fed with standard pellet diet with free access to food and water. Adult male mice (4–8 months) were then euthanized under anesthesia via cardiac perfusion with phosphate-buffered saline (PBS; pH 7.6), and the peripheral tissues were cleaned from blood contaminants. The testes were rapidly removed and stored at −80°C for molecular investigations and/or fixed in Bouin’s solution for morphological analyses or direct use in *ex vivo* treatment with EX527 (6-chloro-2,3,4,9-tetrahydro-1H-carbazole-1-carboxamide), as described below. The number of the animals enrolled was determined by the parameters established through the G*Power analysis (ver.3.1.9.7; Heinrich-Heine-Universitat Dusseldorf, Dusseldorf, Germany; http://www.gpower.hhu.de/) required to obtain permission for *in vivo* experiments in Italy, as suggested by the Legal Entity sanctioning such permission. All experiments involving animals were approved by the Italian Ministry of Education and Italian Ministry of Health (authorization no. 48/2022-PR). The procedures involving animal care were carried out in accordance with the National Research Council’s Guide for Care and Use of Laboratory Animals (National of Institutes of Health Guide).

### *Ex vivo* treatment with EX527

EX527 is a potent and selective inhibitor of SIRT1 activity that was purchased from Abcam (cat. no. ab141506, Cambridge, United Kingdom). The drug was dissolved in dimethylsulfoxide (DMSO) according to the manufacturer’s instructions. Cb1^−/−^ testes (n = 4/experimental group) were feebly punctured in the tunica albuginea and incubated in 6 mL of PBS for 90 min at room temperature (RT) with the vehicle (0.005% of DMSO; control group, CTRL) or in 10 μM of EX527. DMSO was then added at a final concentration of 0.005% to each experimental group. After treatment, the testes were stored at −80°C for protein extraction or fixed in Bouin’s solution for morphological investigations.

### *In vivo* experiment with 17-β estradiol (E_2_)

Prepubertal Cb1^−/−^ male mice were treated with E_2_± ICI (i.e., ICI182780, which is a potent estrogen receptor antagonist) from 24 to 70 days postpartum (dpp), as reported previously (Cacciola et al., 2013b). Briefly, 24-day-old Cb1^−/−^ mice (n = 12) were organized into three experimental groups (n = 4 animals/group) and intraperitoneally injected with 100 µL of physiological solution (0.9% NaCl) containing the vehicle (1% ethanol) or E_2_ (1.5 μg/100 g) or E_2_ + ICI (1.5 μg E_2_/100 g + 15 μg ICI/100 g) for 6 weeks on alternate days. Each dose of E_2_ and E_2_+ICI contained 1% ethanol, similar to the vehicle dose. At the end of treatment, all the animals, including untreated 70-day-old WT mice used herein as the controls, were sacrificed as described above. The testes were rapidly removed and properly stored depending on the experimental procedure. At least three testes/experimental group were used for the protein extraction procedures. Drug treatments were performed on 24 dpp mice during the first wave of spermatogenesis, when the presence of round SPTs in the seminiferous tubule was ascertained, and stopped 6 weeks later at the age of 70 dpp. This time window was chosen to ensure completion of spermiohistogenesis and maturation of the SPZs.

### Protein extraction and Western blot (WB) analysis

The testes collected from the WT Cb1^+/−^ and Cb1^−/−^ mice (n = 4/genotype) or relative to the *ex vivo* and *in vivo* treatments (n = 4 samples for each experimental group) were homogenized in triple detergent RIPA buffer [containing PBS at pH 7.4, 10 mM of dithiothreitol, 0.02% of sodium azide, 0.1% of SDS, 1% of NP-40, 0.5% of sodium deoxycholate, and protease inhibitors (10 μg/mL of leupeptin, aprotinin, pepstatin A, chymostatin, and 5 μg/mL of TPCK)] to obtain the total protein lysates for WB analysis, as described previously (Chioccarelli et al., 2020b). The amount of protein was assessed using the Lowry assay. Equal protein amounts were separated by SDS-PAGE (9% acrylamide or 4%–20% acrylamide gradient) and transferred to polyvinylidene difluoride membranes (GE Healthcare) at 280 mA for 2.5 h at 4°C. The membranes were cut depending on the experimental needs. Individual WB analyses were performed for each antibody to avoid signal alterations dependent on membrane stripping. The antibodies and relative dilutions are reported in Table 1. The signals and background intensity were quantified by densitometry analysis using ImageJ software, adjusted relative to ERK-2 or total histone H3, and graphed in terms of optical density (OD) values as fold changes (mean ± standard error of the mean (SEM)).

**TABLE 1:** Primary antibodies used in the Western blot (WB) and immunohistochemistry (IHC)/immunoprecipitation (IP) analyses.

Primary antibody	Dilution	Provider (catalog number)
SIRT1	1:1,000 (WB) 1:100 (IHC)	Abcam (ab110304), United Kingdom
MOF	1:500(WB) 2 μg (IP)	Sant Cruz (sc-81163), TX, United States
Total histone H3	1:1,000(WB)	Sigma Aldrich (05-928), Milan, Italy
Acetyl histone H4 epitope H4-K5ac/K8ac/K12ac/K16ac(H4tetraAc)	1:1,000 (WB) 1:100 (IHC)	Sigma Aldrich (05-1355), Milan, Italy
Acetyl histone H4 K5	1:1,000(WB)	Elabscience (E-AB-20207), Houston, TX, United States
Acetyl histone H4 K8	1:1,000(WB)	Elabscience (E-AB-20208), Houston, TX, United States
Acetyl histone H4 K12	1:1,000(WB)	Elabscience (E-AB-20209), Houston, TX, United States
Acetyl histone H4 K16	1:1,000 (WB) 1:100 (IHC)	Elabscience (E-AB-20210), Houston, TX, United States
Ac-lysine (AKLC1)	1:500 (WB) 2 μg (IP)	Sant Cruz (sc-32268), TX, United States
ERK-2	1:1,000 (WB)	Sant Cruz (sc-1647), TX, United States
Normal mouse IgG	2 μg (IP)	Sigma Aldrich (12-371), Milan, Italy
Total histone H3 (ChIP grade)	2–5 μg (ChIP)	Abcam (ab1791), United Kingdom

### Immunohistochemistry (IHC) analysis

Testes from the WT Cb1^+/−^ and Cb1^−/−^ mice (n = 4/genotype) or relative to the *ex vivo* treatments (n = 4 samples for each experimental group) were fixed overnight in Bouin’s solution, dehydrated in ethanol, cleared in xylene, and embedded in paraffin using standard procedures. For the IHC analysis, the testis cross-sections (7 µm thick) were deparaffinized, rehydrated, and permeabilized with PBS at pH 7.4 containing 0.1% of Triton-X-100. Next, 0.01 M of a citrate buffer (pH 6.0) was used for antigen retrieval. After blocking with PBS containing 5% of bovine serum albumin (BSA) and normal goat serum (diluted 1:5), the sections were incubated overnight with different primary antibodies (see Table 1) at 4°C. The immunoreactivity was revealed using the avidin/biotin complex system along with H_2_O_2_ and 3,3′-diaminobenzidine-tetrahydrochloride (DAB) as the substrate and chromogen, respectively. For each comparison performed, the testicular cross-sections were placed on the same slide to undergo an equal incubation time with the DAB colorimetric reactive to ensure accuracy of the staining. The reaction specificity was checked by omitting the primary antibody, and red blood cells were used as the internal control of endogenous peroxidase erasing. The histological observations and analyses were conducted under a light microscope (Leica CTR500, Leica Microsystems Inc., Milan, Italy), and the images were captured using a high-resolution digital camera (DC300F; Leica Microsystems Inc., Milan, Italy). For each genotype/experimental group, we analyzed four different samples. At least three technical replicates were assessed for each sample (six sections from different parts of the testis with approximately 30–40 tubules for each section) by two independent blinded observers. The spermatogenic stages VIII, IX and X were observed in approximately 20%–40% of the cross-sectioned tubules. In addition to the IHC quantifications performed by the spermatogenesis experts, color quantification of the DAB colorimetric reaction was performed using ImageJ software (Supplementary Tables S1–S5) on specific regions of interest (ROIs) on black/white converted images captured at the germ cell nuclear level.

### Identification of seminiferous epithelium stages

Seminiferous epithelium stages VIII, IX, and X were identified according to specific staging-identification criteria, including types of spermatocytes (SPCs) present in the basal compartment, sizes and positions of pachytene SPCs, types of SPTs, and orientations of the elongated/condensed SPTs toward the abluminal compartment (Hess, 1999; Hess and de Franca, 2008; Meistrich and Hess, 2013; Xu et al., 2021). The stage VIII was identified by the presence of preleptotene SPCs at the level of the basal compartment along with the presence of round and elongated/condensed SPTs in the adluminal compartment. To discriminate stage VIII from the earlier stages, specific step 16 elongated spermatid parameters were evaluated, including their proximity and orientation in line to the lumen as well as the presence of residual bodies across the spermatid head. The stage IX was identified by the presence of leptotene SPCs at the level of the basal compartment, pachytene SPCs having sizes larger than of those at the previous stages, and elongated SPTs with a peculiar head shape characterized by oblong nuclei that were not yet elongated. Finally, stage X was identified by the presence of larger pachytene SPTs with nuclei reaching their maximum diameter before entering the diplotene phase as well as elongated SPTs characterized by elongating nuclei that were not completely flattened.

### Protein immunoprecipitation (IP)

Protein lysates (25 μg/μL) from at least four testes/genotypes or experimental groups were obtained using triple detergent RIPA buffer, as reported above. To reduce the detergent concentration, the lysates (500 μg) were diluted in PBS (final volume reaction of 1 mL) and separately immunoprecipitated overnight under rotary agitation at 4°C using 2 μg of anti-MOF antibody (see Table 1). Protein A/G PLUS agarose beads (sc-2003; Santa Cruz Biotechnology, Heidelberg, Germany) were added to each sample for 4 h at 4°C under rotary agitation (3,000×*g* for 3 min at 4°C) and then washed thrice in 500 µL of cold Tris-buffered saline (TBS) at pH 7.6. The samples were then boiled in Laemmli sample buffer for 10 min, and the immunoprecipitated proteins were separated by SDS-PAGE and analyzed by WB using different primary antibodies (see Table 1), as described above. The signals were then quantified by densitometry analysis, normalized against the molecular bait MOF signal, and expressed as fold change values. Each experiment was performed in triplicate in comparison to the relative input controls.

### Statistical analysis

Student’s t-test (for comparison of two independent groups) and ANOVA followed by Tukey’s test (for multigroup comparison) were conducted to identify the statistical significance across groups. Differences with *p* < 0.05 were considered to be statistically significant. The data were expressed in terms of mean ± SEM from at least three or four independent animals for each genotype or experimental group. For the WB and IP analyses, triplicate data from each of four animals/genotype or experimental group were considered.

## Results

### Overexpression of SIRT1 in eSPTs is related to H4 hypoacetylation and histone displacement impairment

The *Cb1*-gene deletion reduces the efficiency of histone displacement by negatively affecting the testicular content of H4tetraAc (Chioccarelli et al., 2020b). Interestingly, SIRT1 expression is responsive to CB1 activity in several cell types (Zhang et al., 2018; Azar et al., 2020) and is involved in H4 hyperacetylation underlying histone displacement in eSPTs (Bell et al., 2014). To further investigate the involvement of SIRT1 in the H4 hyperacetylation pathway implicated in histone removal, we took advantage of Cb1^−/−^mouse phenotype (i.e., impaired histone displacement and H4 hypoacetylation) to analyze the SIRT1 and H4tetraAc contents in the testes of WT, Cb1^+/−^, and Cb1^−/−^ mice by WB and IHC analyses. Consistent with our aim, we used a H4tetraAc antibody with selective specificity against H4 tetra-acetylation in -K5/-K8/-K12/-K16 (see database http://www.histoneantibodies.com). Furthermore, we limited the morphological observations to epithelial stages VIII–X of the spermatogenetic cycle by focusing on steps 8–10 eSPTs.

**FIGURE 1:**
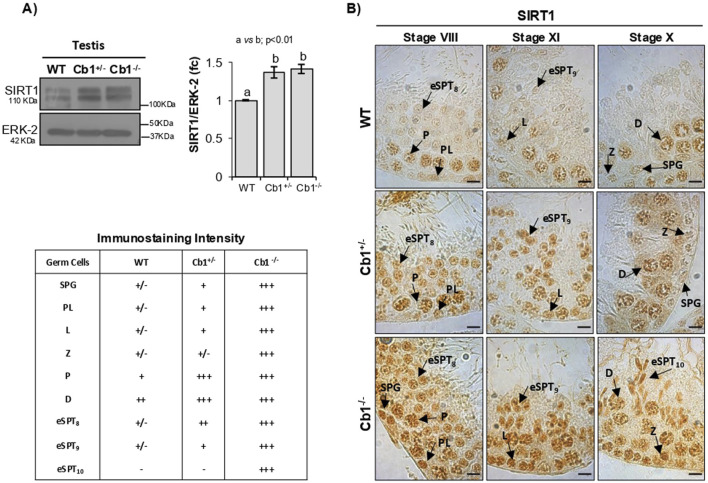
Characterization of SIRT1 in wild type (WT), Cb1^+/−^, and Cb1^−/−^ testes. **(A)** Western blot (WB) analyses of SIRT1 in the WT, Cb1^+/−^, and Cb1^−/−^ testes, where the protein amounts were quantified by densitometry analysis, normalized against ERK-2, and expressed in optical density (OD) values as fold change (fc). All data are reported as mean ± SEM, and experimental groups with statistically significant differences are indicated by different letters. **(B)** Immunohistochemistry (IHC) analysis of SIRT1 in Bouin’s-fixed WT, Cb1^+/−^, and Cb1^−/−^ testes sections (7 μm thick). The black arrowheads indicate localization of SIRT1 in spermatogonia (SPG), preleptotene (PL), leptotene (L), zygotene (Z), diplotene (D), pachytene (P), round spermatids step 8 (rSPT_8_), and elongating spermatids steps 9 and 10 (eSPT_9-10_). Scale bar: 20 μm. The table on the bottom left indicates SIRT1 staining in different germ cells with intensities null (−), light (+/−), weak (+), moderate (++), and strong (+++).

As shown in Figure 1A, the SIRT1 levels were significantly higher in the testes of Cb1^+/−^ and Cb1^−/−^ mice compared to WT. Although SIRT1 was broadly expressed in the germ cells, a higher SIRT1 staining was observed in the germ cells of Cb1^+/−^ and Cb1^−/−^ compared to WT mice from spermatogonia (SPGs) to eSPTs (Figure 1B). In detail, although the more pronounced staining in Cb1^+/−^ was mainly related to leptotene (_L_SPCs), pachytene (_P_SPCs), and diplotene spermatocytes (_D_SPCs), in the Cb1^−/−^ mice it was particularly evident in all germ cell types in the Cb1^−/−^ mice. Interestingly, SIRT1-positive cells overlapped across genotypes except for eSPTs. In the WT testis, SIRT1 staining was weakly present in step 8 eSPTs (stage VIII); this signal was still feebly detectable in step 9 eSPTs (stage IX) and absent in step 10 eSPTs (stage X). No immunostaining was detected in the eSPTs from step 10 onward (data not shown). In the Cb1^+/−^ testis, SIRT1 labeling was observed in step 8 and 9 eSPTs (stages VIII and IX). Despite the staining intensity in these steps was more pronounced than WT counterpart, no immunostaining was detected in step 10 eSPTs, indicating that SIRT1 disappeared in step 10 eSPTs in both the WT and Cb1^+/−^mice (staining details are reported in the left table of Figure 1). In the Cb1^−/−^ testis, SIRT1 was robustly expressed in eSPTs; the staining was intense in step 8 and 9 eSPTs and abnormally retained in step 10 eSPTs, revealing overexpression and anomalous persistence of SIRT1 in these cells.

**FIGURE 2:**
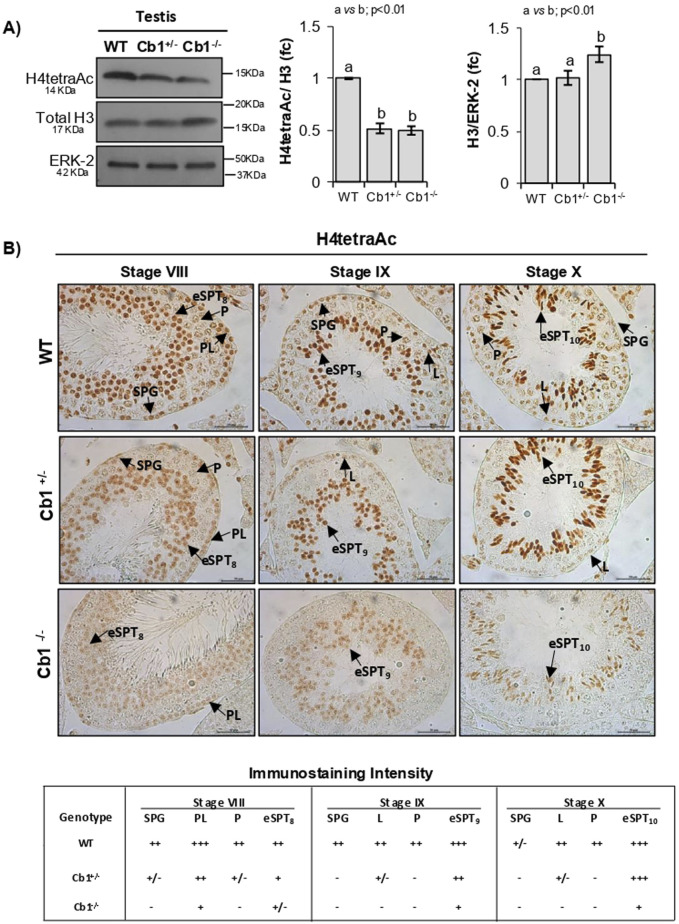
Characterization of H4tetraAc in the WT, Cb1^+/−^, and Cb1^−/−^ testes. **(A)** WB analyses of H4tetraAc and total histone H3 in the WT, Cb1^+/−^, and Cb1^−/−^ testes, where the protein amounts are quantified by densitometry analysis, normalized against ERK-2 or H3/ERK-2, and expressed in OD values as fold change (fc). All data are reported as mean ± SEM, and experimental groups with statistically significant differences are indicated by different letters. **(B)** IHC analysis of H4tetraAc in Bouin’s-fixed WT, Cb1^+/−^, and Cb1^−/−^ testes sections (7 μm thick). The black arrowheads indicate the localization of H4tetraAc in spermatogonia (SPG), preleptotene (PL), leptotene (L), pachytene (P), round spermatids step 8 (rSPT_8_), and elongating spermatids steps 9 and 10 (eSPT_9-10_). Scale bar: 50 μm. The lower table shows H4tetraAc staining in different germ cells with intensities null (−), light (+/−), weak (+), moderate (++), and strong (+++).

According to previously published results (Chioccarelli et al., 2020b), significant deficits of H4tetraAc was observed in Cb1^+/−^ and Cb1^−/−^ testes (Figure 2A). The IHC analysis revealed that H4tetraAc impairment was differently related to germ cells depending on the genotype (Figure 2B). Indeed, while in Cb1^+/−^ mice the H4tetraAc impairment was mainly associated with SPGs and SPCs, in Cb1^+/−^ mice, it was relevant and persistently present also in eSPTs from stages VIII to X. Although H4tetraAc was less moderately enriched in Cb1^+/−^ eSPTs than the WT counterpart, its staining increased progressively in eSPTs from step 8 onward, with a burst in step 10 eSPTs, as similarly observed in WT mice. Conversely, the significant deficit of H4tetraAc observed in Cb1^−/−^ eSPTs from step 8 onward was strikingly evident in step 10 eSPTs (staining details are reported in the lower table of Figure 2). In these cells, the H4 hyperacetylation burst clearly occurred in WT but was absent in Cb1^−/−^ mice. In agreement with these results, the histone removal, herein monitored and further verified by morphological assessment of total histone H3, happened physiologically in heterozygotes and was significantly disrupted in Cb1^−/−^ mice (Supplementary Material S1 and Supplementary Figures S1A,B). In addition, the production of Cb1^−/−^ SPZs with abnormal histone content and abnormally retained chromatin H3-binding sites, was furtherly confirmed by Chromatin immunoprecipitation sequencing (ChIP-seq) experiments, (Supplementary Material S2 and Supplementary Figures S2A–C), suggesting that the anomalous persistence of SIRT1 in Cb1^−/−^ step 10 eSPTs disrupted histone displacement presumably via the impairment of histone H4 acetylation burst.

### SIRT1 persistence in eSPTs is related to K16-based H4 hypoacetylation

**FIGURE 3:**
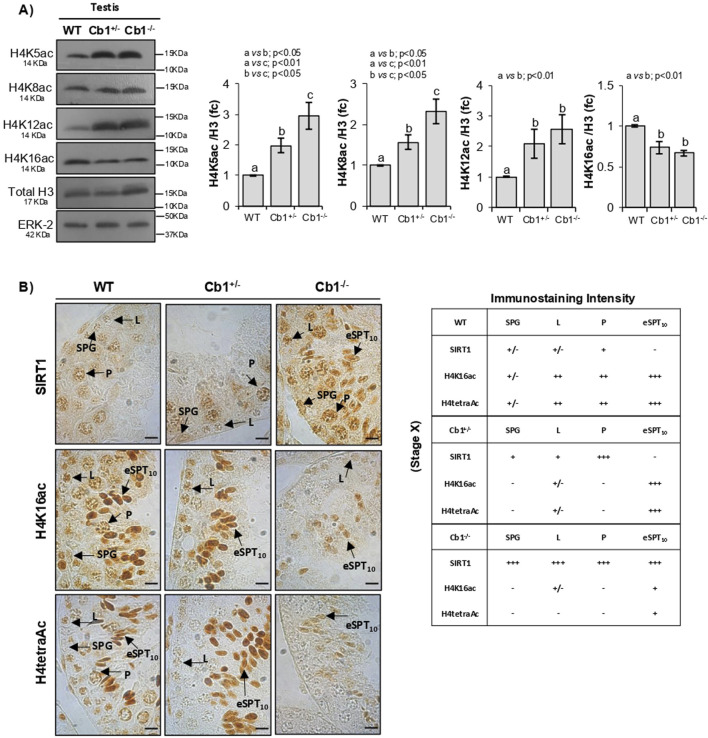
Characterization of H4-monoacetylated forms related to H4tetraAc deficit in step10 eSPTs overexpressing SIRT1. **(A)** WB analyses of H4K5Ac, H4K8Ac, H4K12Ac, and H4K16ac in the WT, Cb1^+/−^, and Cb1^−/−^ testes, where the protein amounts are quantified by densitometry analysis, normalized against H3/ERK-2 signals, and expressed in OD values as fold change (fc). All data are reported as mean ± SEM, and experimental groups with statistically significant differences are indicated by different letters. **(B)** IHC analyses of SIRT1, H4K16ac, and H4tetraAc in Bouin’s-fixed WT, Cb1^+/−^, and Cb1^−/−^ testes sections (7 μm thick). The localization of different signals in spermatogonia (SPG), leptotene (L), pachytene (P), and elongating spermatids step 10 (eSPT_10_) are indicated by black arrowheads. Scale bar: 20 μm. The table on the bottom right indicates SIRT1, H4K16ac, and H4tetraAc staining in different germ cells with intensities null (−), light (+/−), weak (+), moderate (++), and strong (+++).

To identify which of the four lysines implicated in the H4 hyperacetylation burst was subject to hypoacetylation, we analyzed the monoacetylated forms H4-K5ac, -K8ac, -K12ac, and -K16ac in the WT, Cb1^+/−^, and Cb1^−/−^ testes by WB. The results reveled significant enrichments of H4K5ac, H4K8ac, and H4K12ac in the testes of Cb1^+/−^ and Cb1^−/−^ mice compared to WT (Figure 3A). However, the H4K5ac and H4K8ac contents were higher in the testes of the Cb1^−/−^ vs. Cb1^+/−^ mice. Conversely, H4K16ac was significantly lower in the testes of Cb1^+/−^ and Cb1^−/−^mice than WT, well explaining the impairment of H4tetraAc reported in the germ cells, especially in the eSPTs and/or SPCs depending on genotype.

To investigate the emerging link between SIRT1 expression and H4K16ac enrichment related to H4 hyperacetylation in the eSPTs, we focused on stage X of the epithelial cycle and compared the expression of SIRT1 with the enrichments of H4K16ac and H4tetraAc in step 10 eSPTs of the WT, Cb1^+/−^, and Cb1^−/−^ mice by IHC. The results confirmed the absence of SIRT1 in step 10 eSPTs both in WT and Cb1^+/−^ mice (Figure 3B). As expected, the absence of SIRT1 was associated with relevant enrichments of H4K16ac and H4tetraAc in the same cells. Conversely, the persistence of SIRT1 in step 10 eSPTs of the Cb1^−/−^ mice was associated with feeble enrichments of H4K16ac and H4tetraAc (staining details are reported in the right table). Considering the relevant accumulations of H4K5ac, H4K8ac, and H4K12ac observed in step 10 eSPTs of the Cb1^−/−^ vs*.* WT mice (Supplementary Figure S3), our results strongly suggest a causal link between abnormal SIRT1 persistence and H4 hyperacetylation burst failure, likely dependent on the hypoacetylation of H4 at lysine K16.

### SIRT1 inactivation in Cb1^−/−^ testis promotes MOF-dependent H4K16ac-based hyperacetylation of histone H4 and restores histone displacement

SIRT1 has been reported to interact with and regulate the histone acetyltransferase MOF by affecting its lysine acetylation, stability, and activity in both *in vitro* and *in vivo* systems (Peng et al., 2012; Lu et al., 2011). Importantly, MOF is the major H4K16-acetyltransferase characterized in eSPTs (Lu et al., 2010). Therefore, we hypothesized that the abnormal SIRT1 retention could affect and deregulate MOF through direct or indirect SIRT1/MOF interaction downstream impairing H4K16 acetylation and related H4 hyperacetylation burst in step 10 eSPTs.

**FIGURE 4:**
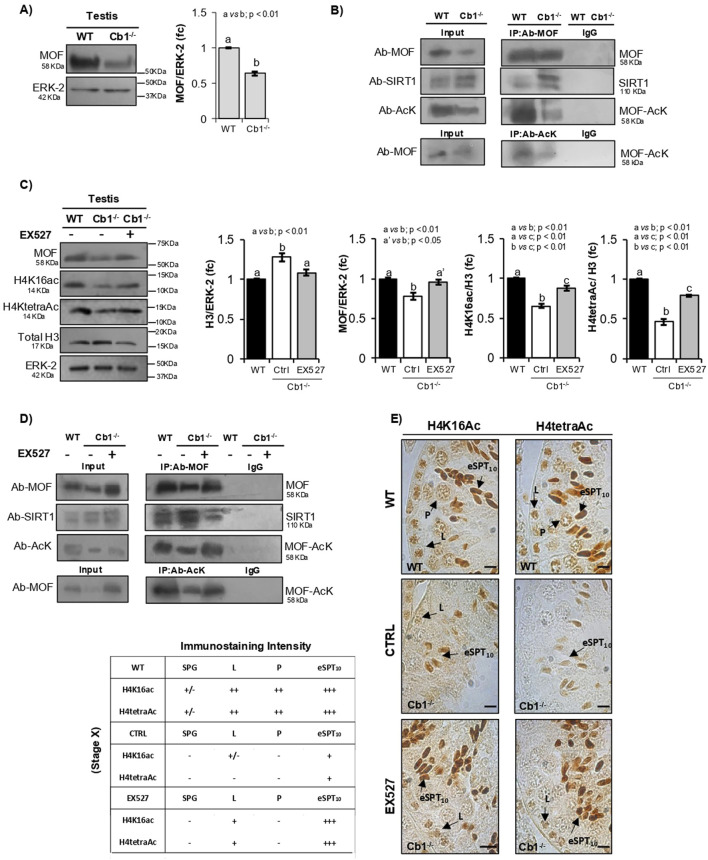
Characterization of MOF/SIRT1 interaction in the pathway of H4 hyperacetylation responsive to SIRT1 activity and inhibition. **(A)** WB analysis of MOF in the WT and Cb1^−/−^ testes. All signals are quantified by densitometry analysis and normalized against ERK-2; the data are expressed in OD values as fold change (fc) and reported as mean ± SEM. Experimental groups with statistically significant differences are indicated by different letters. **(B)** Total proteins from WT and Cb1^−/−^ testes were immunoprecipitated using MOF antibody, AcK antibody, or pre-immune IgG. The precipitates were immunoblotted with anti-MOF, anti-SIRT1, and anti-AcK antibodies. **(C)** WB analyses of MOF, H4K16ac, H4tetraAc, and total H3 in the WT and Cb1^−/−^ testes treated *ex vivo* with vehicle (CTRL) or EX527. The protein amounts are quantified by densitometry analysis, normalized against ERK-2 or H3/ERK-2 signals, and expressed in OD values as fold change (fc). All data are reported as mean ±SEM, and experimental groups with statistically significant differences are indicated by different letters. **(D)** Total proteins from the WT and Cb1^−/−^ testes treated *ex vivo* with vehicle (CTRL) or EX527 were immunoprecipitated using MOF antibody, AcK antibody, or pre-immune IgG. The precipitates were immunoblotted with anti-MOF, anti-SIRT1, and anti-AcK antibodies. **(E)** IHC analyses of H4K16ac and H4tetraAc in Bouin’s-fixed sections (7 μm thick) of WT and Cb1^−/−^ testes treated *ex vivo* with vehicle (CTRL) or EX527. The black arrowheads indicate the localization of H4K16ac and H4tetraAc in spermatogonia (SPG), leptotene (L), pachytene (P), and elongating spermatids step 10 (eSPT_10_). Scale bar: 20 μm. The table on the bottom left indicates H4K16ac and H4tetraAc staining in different germ cells with intensities null (−), light (+/−), weak (+), moderate (++), and strong (+++).

To assess our hypothesis, we excluded the Cb1^+/−^ testis from our analysis since histone displacement occurs efficiently in Cb1^+/−^ mice and investigated the MOF protein contents in the testes of WT and Cb1^−/−^ mice by WB. To verify the MOF/SIRT1 interactions and MOF acetylation status, we immunoprecipitated the WT and Cb1^−/−^ testis lysates with anti-MOF antibody and probed the immunocomplexes with anti-MOF, -SIRT1, and -acetyl lysine (AcK) antibodies by WB (Figure 4B). We verified that the anti-AcK antibody recognized a protein with a whose molecular weight overlapping that of MOF in order to analyze the acetylated form of the MOF protein (MOF-AcK). To further analyze MOF-AcK, we also immunoprecipitated WT and Cb1^−/−^ testis lysates with anti-AcK antibody and probed the immunocomplexes with anti-MOF antibody.

As reported in Figure 4A, a significantly lower content of MOF protein was observed in testis of Cb1^−/−^ vs*.* WT mice. In agreement, IP confirmed the observed reduction of MOF content in the Cb1^−/−^ testis (Figure 4B). The MOF/SIRT1 co-precipitation clearly revealed that MOF was directly or indirectly able to interact with SIRT1 in both the WT and Cb1^−/−^ testes. The MOF/SIRT1 interaction was stronger in Cb1^−/−^ than WT testis lysates. In parallel, the MOF-AcK content was strongly reduced in Cb1^−/−^ compared to WT (Supplementary Figures S4A,B). This suggested that the abnormal MOF/SIRT1 ratio disrupted the chain of molecular events involved in H4 hyperacetylation by enhancing SIRT1-driven MOF hypoacetylation.

To confirm our hypothesis, we attempted to reverse this phenotype using Cb1^−/−^ testis cultured *ex vivo* with a potent and selective SIRT1 inhibitor (EX527) as an experimental strategy. The testes of Cb1^−/−^ mice were *ex vivo* treated with the vehicle (CTRL group) ± 10 μM of EX527 (EX527-treated group) and systematically analyzed in comparison with WT testis, here used here as the control reference. Protein lysates were prepared and used to verify the levels of H3, MOF, H4K16ac, and H4tetraAc by WB analysis. The protein lysates were also immunoprecipitated using anti-MOF or anti-AcK antibodies before being probed for the immunocomplexes, probed as detailed above. To verify the effects of SIRT1 inhibition on the enrichments of H4K16ac and H4tetraAc in step 10 eSPTs, testicular sections from the WT, CTRL, and EX527-treated groups were analyzed by IHC and the epithelial stage X was systematically considered. As expected, the histone H3 content rescued to WT values after EX527 treatment, highlighting the complete recovery of histone displacement in response to SIRT1 inactivation (Figure 4C). In agreement, the MOF, H4K16ac, and H4tetraAc contents increased significantly in the EX527-treated group than the CTRL one, although H4K16ac and H4tetraAc did not fully recover to the WT values. The IP assay confirmed complete recovery of the MOF levels after EX527 treatment (Figure 4D). In parallel, MOF/SIRT1 co-precipitation decreased while the MOF-acetylated form increased significantly (Supplementary Figures S4C,D).

Coherently with the observed rescue of histone displacement, IHC analysis confirmed enrichments of H4K16ac and H4tetraAc in the Cb1^−/−^ step 10 eSPTs after EX527 treatment (Figure 4E). In particular, the staining intensities of H4K16ac and H4tetraAc were higher in step 10 eSPTs of the EX527-treated Cb1^−/−^ mice than the CTRL group and were comparable to the WT one (staining details are reported in the left table in Figure 4). This rescue activity of EX527 on H4K16ac and H4tetraAc in step 10 eSPTs of the Cb1^−/−^ mice highlighted the key role of H4K16 acetylation in H4 hyperacetylation burst responsive to SIRT1 inactivation. Collectively, the data demonstrated that inhibition of SIRT1 activity in the Cb1^−/−^ testis promoted the rescue of histone displacement accomplished by the burst of H4K16ac-based H4TetraAc in step 10 eSPTs. These latter events appeared to be dependent on the recovery of the MOF acetylation state. Interestingly, weak enrichments of H4K16ac and H4tetraAc were evidenced in Cb1^−/−^_L_SPCs after EX527 treatment, whereas no effects were observed in Cb1^−/−^ SPGs and _P_SPCs. These results adequately explained the small deficits of H4K16ac and H4tetraAc detected by WB in the testes of EX527-treated Cb1^−/−^ mice vs*.* WT and interestingly evidenced the existence of a more complex pathway likely responsive to the CB1 receptor to modulating H4 acetylation in these cell types.

### SIRT1 downregulation in Cb1^−/−^ testis restores H4 hyperacetylation pathway eliciting histone displacement

**FIGURE 5:**
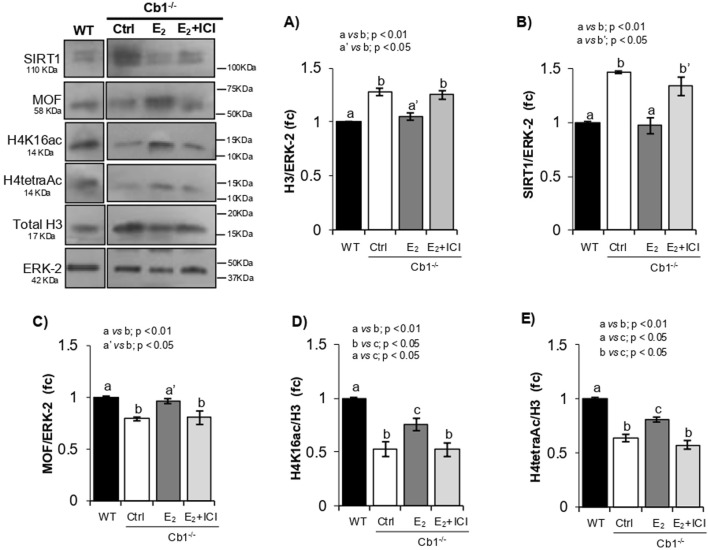
Responsiveness of SIRT1, MOF, H4K16ac, and H4tetraAc to histone displacement recovery. **(A–E)** WB analyses of H3, SIRT1, MOF, H4K16ac, and H4tetraAc in the WT and Cb1^−/−^ testes treated *in vivo* with vehicle (CTRL) or E_2_ ± ICI. All signals are quantified by densitometry analysis against ERK-2 or H3/ERK-2, and the data are expressed in OD values as fold change (fc) and reported as mean ± SEM. The experimental groups with statistically significant differences are indicated by different letters.

Cb1^−/−^ male mice injected with low-dose E_2_ respond to treatment by fully recovering histone displacement (Cacciola et al., 2013b; Chioccarelli et al., 2020b). Thus, E_2_-treated Cb1^−/−^ mice represent a useful “*in vivo* the model” for reestablishing the physiological histone removal mechanism and validating the *in vivo* the relationship between SIRT1 level and H4 hyperacetylation pathway underlying histone displacement. Cb1^−/−^ male mice were injected with the vehicle (CTRL) or E_2_ ± ICI, and the testicular levels of SIRT1, MOF, H4K16ac, and H4tetraAc were analyzed by WB. The efficiency of histone displacement in these subjects was assessed through measurement of the testicular content of histone H3 (Figure 5A).

As expected, SIRT1 and MOF responded inversely to treatment. In agreement with the recovery of histone displacement, a significant decrease in SIRT1 and concomitant increase in MOF contents were observed in the testes of E_2_-treated Cb1^−/−^ mice compared to the CTRL- and E_2_+ICI-treated testes (Figures 5B,C). Although both proteins showed comparable levels in the CTRL and E2+ICI groups, the MOF protein increased to physiological values after E_2_ treatment in response to SIRT1 downregulation, coherently with the enrichments of H4K16ac and H4tetraAc as well as recovery of histone displacement (Figures 5D,E). Therefore, the inverse responsiveness of SIRT1 and MOF to histone displacement recovery, accomplished to the increase of H4K16ac and H4tetraAc, validated their causal associations in modulating the H4 hyperacetylation burst implicated in histone removal. Despite these enrichments, small deficits of H4K16ac and H4tetraAc were observed in the testes of E_2_-treated Cb1^−/−^ mice compared to WT. The histone displacement recovery observed in E_2_-treated Cb1^−/−^ mice suggests that this deficit was not related to the eSPTs but rather to other germ cells, probably SPGs and SPCs, as demonstrated by SIRT1 inhibition in the *ex vivo* experiments. Hence, further studies are necessary to clarify this aspect.

## Discussion

In the recent past, it has been hypothesized that sperm histone-based chromatin plays a pivotal role in embryonic development and in determining the onset of disease in adulthood (Jung et al., 2017). In this context, mechanistic knowledge of the pathways underlying histone displacement in eSPTs has become particularly relevant. In this study, taking advantage of the phenotype of Cb1^−/−^ mouse phenotype (Chioccarelli et al., 2010; Chioccarelli et al., 2020b), we investigated the role of SIRT1 in H4 hyperacetylation mechanism driving histone displacement in eSPTs (Hazzouri et al., 2000; Ketchum et al., 2018). We show that the hyperacetylation of histone H4 occurs progressively in step 8 and 9 eSPTs but bursts in step 10 SPTs, appearing inversely correlated to the expression pattern of SIRT1 in these cells. Indeed, SIRT1 was scantly present in step 8, weakly detectable in step 9, and absent in step 10 eSPTs, hinting at the possibility that the H4 hyperacetylation burst was responsive to SIRT1 disappearance. The anomalous retention of SIRT1 and the notable reduction of H4tetraAc, both observed in step 10 eSPTs of Cb1^−/−^ mice in combination with the enrichment of H4K5ac, H4K8ac, and H4K12ac and the under acetylation of H4K16, are consistent with this idea suggesting that theH4K16ac enrichment in eSPTs exerts a key role in H4 hyperacetylation burst implicated in histone displacement (Ketchum et al., 2018).

Studies carried out in germ-cell-specific *Sirt1* knockout mice reveal that SIRT1 is required for H4 acetylation in lysines K5, K8, and K12 (Bell et al., 2014). These acetylation tags decrease in response to *Sirt1* deletion (Bell et al., 2014), and the data reported in this study coherently show their responsiveness to SIRT1 overexpression. Contrarily, *Sirt1* deletion in the germ cells does not affect H4K16ac content (Bell et al., 2014), demonstrating that H4K16 acetylation occurs efficiently in the absence of SIRT1 and that H4K16ac is not a direct target of SIRT1 in germ cells. It is worth noting that H4K16ac reportedly enriches eSPTs at the end of nuclear elongation (Hazzouri et al., 2000; Ketchum et al., 2018). We show that such enrichment is responsive to SIRT1 disappearance in step 10 eSPTs. Indeed, the absence of SIRT1 in these cells is critically relevant for H4K16ac accumulation because SIRT1 directly or indirectly interacts and downregulates the H4K16-acetyltransferase MOF in terms of acetylation status and protein content, thus providing the plausible mechanism by which H4K16ac efficiently enriches eSPTs when SIRT1 disappears in step 10 (Lu et al., 2010; Sin et al., 2012; Chioccarelli et al., 2020a). In agreement, the overexpression of SIRT1 in human somatic cells has been reported to reduce protein levels of hMOF. Mechanistically, SIRT1 binds and deacetylates hMOF; the SIRT1/hMOF interaction involves the catalytic domains of both enzymes and promotes deacetylation of the MYST enzymatic domain of hMOF with negative effects on hMOF stability, autoacetylation, and HAT activity (Peng et al., 2012). It is therefore reasonable that the persistence of SIRT1, noted here in the step 10 eSPTs of Cb1^−/−^ mice, impairs the accumulation of H4K16ac by negatively affecting MOF protein stability rather than its expression. Further investigations are needed to better define these aspects. However, we show that counteracting the abnormal MOF/SIRT1 interactions in Cb1^−/−^ testes by SIRT1 inhibition can increase MOF protein content and its acetylation status, increased, downstream promoting the full recovery of H4K16ac in step 10 eSPTs downstream and thereby confirming the responsiveness of the H4K16 acetylation pathway to SIRT1 activity. The marked enrichments of H4K5ac, H4K8ac, and H4K12ac observed in these cells, along with the last acetyl group added to the K16 of histone H4 in response to SIRT1 inhibition or downregulation, restore the H4 hyperacetylation burst as well as histone removal in Cb1^−/−^ mice. These results confirm the critical involvement of the H4K16 acetylation tag in the histone displacement mechanism (Chioccarelli et al., 2020a). Noteworthy, neither the acetylation of H4 on lysines -K5, -K8, and -K12 (data reported herein) nor acetylation of H4 on lysine K16 alone (Bell et al., 2014) are sufficient to promote nucleosome eviction. Although the exact role of hyperacetylated H4 in this mechanism and the contributions of each of the acetylation tag are currently unclear, it is known that lysine acetylation weakens the DNA–histone interaction, counteracts the interactions between nucleosomes, and loosens the nucleosome clutches, leading to chromatin relaxation and unfolding (Chioccarelli et al., 2020a; Martinez et al., 2023; Portillo-Ledesma et al., 2021). Complementary to such structural significance, histone acetylation provides binding signals for the recruitment of acetyl-lysine readers and chromatin remodeling complexes (Chioccarelli et al., 2020a). Interestingly, BRDT can recognize the di- (K5/K8) and tetra- (K5/K8/K12/K16) acetylated H4 tails with equal affinities (Dhar et al., 2012); further, the BRDT-SMARCE1 interaction increases both *in vitro* and *in vivo* following H4 hyperacetylation (Dhar et al., 2012). H4K16 acetylation is known to uniquely inhibit the interaction between the N-terminal tail of H4 and the “acid patch” on the surface of the neighboring nucleosome (Kalashnikova et al., 2013). This suggests the plausible mechanisms by which the first acetylations at H4-K5/K8/K12 could promote chromatin fiber relaxation and BRDT binding, making chromatin prone to displace histones. The last acetylation added on H4 at lysine K16, in addition to increasing chromatin unwinding, could expose the surface of hyperacetylated nucleosomes with the effect of higher tethering of SMARCE1 to chromatin. The resulting increase in BRDT-SMARCE1 interaction could then initiate displacement of the acetylated histones. Coherently with this hypothesis and in agreement with the major wave of chromatin opening recently characterized by ATAC-seq in eSPTs prior to histone displacement (Luense et al., 2019), it is known that H4K16ac can reduce intra- and inter-chromatin fiber interactions (Shogren-Knaak et al., 2006), promote mobility of the neighboring nucleosome (Kalashnikova et al., 2013), and enhance exposure of the nucleosome surface tethering domain to acid-patch binding proteins, including chromatin remodeling complexes (Kalashnikova et al., 2013; McGinty and Tan, 2021).

**FIGURE 6:**
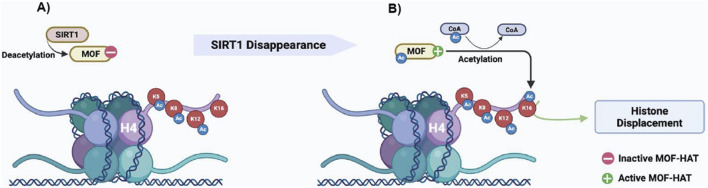
Proposed molecular mechanism of **(A)** SIRT1-mediated MOF deacetylation block MOF-HAT activity toward the lysine 16 of histone H4; **(B)** disappearance of SIRT1 in step 10 eSPTs favors the active acetylated form of MOF, which in turn promotes H4K16ac needed for the start of histone displacement.

Collectively, we provide compelling evidence that H4K16ac and H4tetraAc enrichments co-occur efficiently when SIRT1 disappears in the step 10 eSPTs. The persistence of SIRT1 in these cells interferes with H4tetraAc by reducing MOF-dependent H4K16 acetylation (Figure 6). Although we do not know whether directly or indirectly, SIRT1 interacts with and deacetylates MOF, resulting in H4K16 hypoacetylation, impaired histone removal and enrichment of sperm histone chromatin fraction. Interestingly, somatic cells that activate DNA repair mechanisms activate the same H4K16 acetylation pathway reported herein (Peng et al., 2012). In somatic cells, SIRT1 is directly involved in DNA repair; however, through a transient and dynamically regulated protein–protein interaction, it also acts indirectly in DNA repair by maintaining critical levels of active H4K16-acetyltransferase hMOF (Peng et al., 2012). These results are in agreement with our findings and underscore the key involvement of the SIRT1-mediated H4K16 acetylation pathway in chromatin remodeling mechanisms underlying histone-to-protamine exchange. Notably, histone-protamine exchange requires the coordinated occurrence of DNA repair and histone displacement (Okada, 2022), suggesting the potential coordinating role of H4K16ac in this regard. In agreement, the histone displacement recovery in Cb1^−/−^ testis reported herein as responsive to the rescue of the H4K16 acetylation pathway in eSPTs of E_2_-treated Cb1^−/−^ mice is also associated with the recovery of sperm DNA integrity, as demonstrated previously (Chioccarelli et al., 2010; Cacciola et al., 2013b, 2013c).

The results presented herein are particularly relevant in the light of the suggested role of the chromatin region retaining the modified or unmodified histones, consisting in promoters of developmental loci and master regulatory genes, including housekeepings, miRNAs, developmental and paternally-expressed imprinted genes (Hammoud et al., 2009; Jung et al., 2017; Yamaguchi et al., 2018). Indeed, anomalies in the sperm histone-chromatin fraction appear to be linked to poor embryogenesis and developmental defects in the offspring (Hammoud et al., 2011; Gaspa-Toneu and Peters, 2023; Dodd and Luense, 2024). Consequently, any interference with histone displacement in eSPTs may interfere with the histone content and epigenome of SPZs with potential transgenerational implications. Notably, SIRT1 is implicated in a plethora of biological mechanisms (Wu et al., 2022); therefore, the responsiveness of SIRT1 to CB1 reported here in the germ cells and already observed in somatic cells suggests caution in the use of cannabis.

The findings of this study motivate several new questions that remain unanswered. We demonstrate that loss of CB1 leads to production of SPZs with abnormal histone-chromatin fractions. In line with the observations that such nucleohistone fractions mark gene loci crucial for chromatin architecture and transcriptional regulation in preimplantation embryos (Hammoud et al., 2011; Gaspa-Toneu and Peters, 2023; Dodd and Luense, 2024), our data support a novel but key role of the Endocannabinoid System (ECS) in the epigenetic modulation of sperm chromatin, still entirely unexplored. The RNA-seq transcriptomic assessments of testes treated *ex vivo* with SIRT1 inhibitor or derived from E_2_-injected mice could contribute to better understand the epigenetic mechanisms modulated by SIRT1/MOF axis.

Finally, an intriguing aspect emerges from the impaired H4 acetylation observed in meiotic cells in response to SIRT1 inhibition. MOF participates in multiple protein complexes, including the male-specific lethal (MSL) and non-specific lethal (NSL) complexes (Li et al., 2010; Dias et al., 2014; Ravens et al., 2014), where specific subunits (mainly MSL1 and NSL1) regulate MOF-HAT activity (Smith et al., 2005; Cai et al., 2010). It is conceivable that the failed recovery of MOF-mediated H4 acetylation in meiotic cells, could be due to the absence of specific MOF cofactors responsive to CB1. Addressing these issues will be a tough challenge.

## Data Availability

The raw data supporting the conclusions of this article will be made available by the authors without undue reservation.
