# Supplementary material for: SIRT1 retention in elongating spermatids interferes with histone displacement by counteracting MOF-dependent H4K16 acetylation
Source: Front Cell Dev Biol. 2025 Aug 29;13:1524919. doi: 10.3389/fcell.2025.1524919 (PMC12426168; doi:10.3389/fcell.2025.1524919)
Supplement: Supplementary file 1 [file DataSheet2.PDF]

Supplementary Material

Supplementary Data: the impairment of histone displacement leads to H3-enriched sperm chromatin

Chromatin immunoprecipitation sequencing (ChIP-seq) experiments using the anti-H3 antibody were performed in WT and $Cb1^{-/-}$ SPZ collected from *caput* epididymis to corroborate the abnormal histone retention characterized in $Cb1^{-/-}$ mice. The *caput* SPZ were chosen as accurately reflects the outcomes of histone displacement events occurring in the testis (Fig. S 3A). We excluded $Cb1^{+/-}$ SPZ from this analysis since histone displacement efficiently occurs in $Cb1^{+/-}$ mice.

Chip-Seq analysis revealed significant differences in the number of H3-marked chromatin regions between WT and $Cb1^{-/-}$ *caput* SPZ. In particular, the analysis of H3 signals showed a total of 1335 H3-associated peaks consisting in 180 and 1176 peaks, respectively detected in WT and $Cb1^{-/-}$ *caput* SPZ, and 20 peaks relatively to gene loci shared by both genotypes (Fig. S 3B). Such difference in the peak number appeared consistent with the increased H3 amount previously characterized in $Cb1^{-/-}$ *caput* SPZ (Chioccarelli et al., 2020b), demonstrating that the defective histone displacement in $Cb1^{-/-}$ mice promoted the production of SPZ with abnormally retained chromatin H3-binding sites. Moreover, the accurate description of H3 signals detected in WT and $Cb1^{-/-}$ *caput* SPZ revealed that the identified peaks localized in different genomic regions with differences in genic (exons, introns and TSS) and in intergenic genomic location. Nearly 80% of H3-associated peaks were localized in the genic regions in WT *caput* SPZ, while approximately 70% of them were localized in the genic regions of $Cb1^{-/-}$ counterpart. Conversely, a reduced population of peaks was localized in intergenic regions of WT *caput* SPZ in comparison to that found in $Cb1^{-/-}$ counterpart (Fig. S 3C).

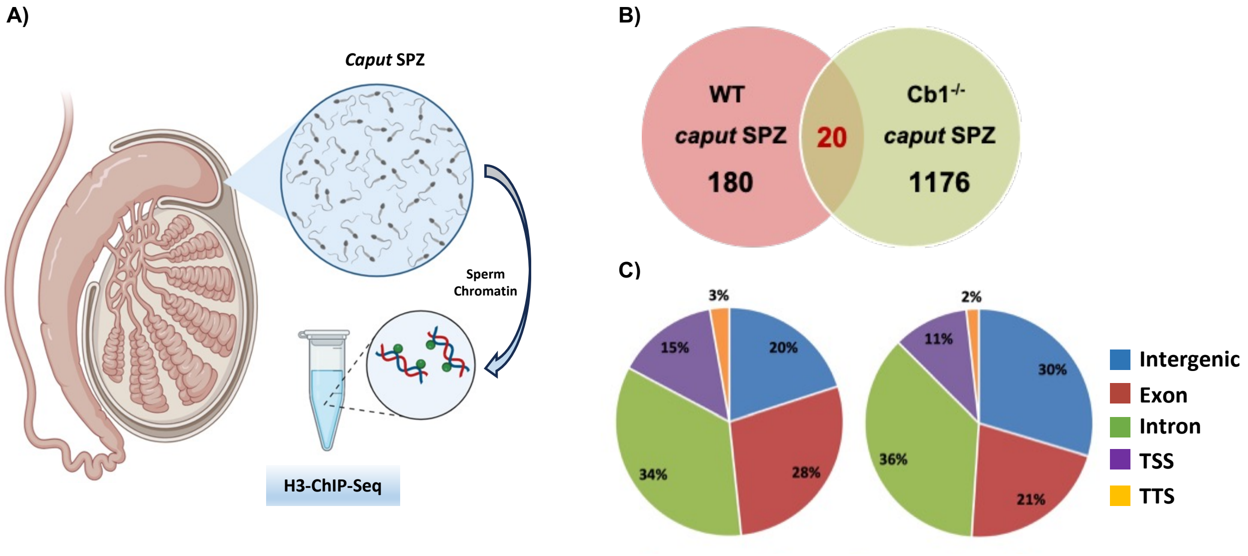

Figure S2. (**A**) Schematic representation of ChIP-seq analysis in WT and Cb1$^{-/-}$ *caput* SPZ. (**B**) Venn Diagram showing 180 chromatin peaks associated with H3 for WT *caput* SPZ, 1176 peaks for Cb1$^{-/-}$ *caput* SPZ and 20 peaks shared by both genotypes. (**C**) Pie chart analysis of separate samples showing the distribution of H3 peaks detected in WT and Cb1$^{-/-}$ *caput* SPZ among various genomic regions.
