# Supplementary material for: SIRT1 retention in elongating spermatids interferes with histone displacement by counteracting MOF-dependent H4K16 acetylation
Source: Front Cell Dev Biol. 2025 Aug 29;13:1524919. doi: 10.3389/fcell.2025.1524919 (PMC12426168; doi:10.3389/fcell.2025.1524919)
Supplement: Supplementary file 2 [file DataSheet4.PDF]

Supplementary Material

Supplementary Methods

Sperm collection

Caput epididymis (from n=5 WT and n=5 $Cb1^{-/-}$) were separately immersed in PBS (pH 7.6) and cut to let SPZ flow out from the ducts. Sperm suspensions were then filtered throughout cheesecloth to eliminate fragments of epididymal tissue and centrifuged at 2000 x g for 30 min at 4°C. After centrifugation, an aliquot of samples was analysed under a light microscope (Leica CTR500, Leica Microsystems Inc., Milan, Italy) to verify possible somatic cell contamination and to assess the amount of not-viable SPZ using Trypan-blue dye (0.4% solution, 17-942E, Lonza, Basel, Switzerland) (data not shown). Then, the SPZ pellets were incubated on ice for 30 min with Somatic Cell Lysis Buffer (SCLB; 0.1% SDS, 0.5% Triton X-100 in DEPC-H2O) to eliminate somatic cells and not-viable SPZ contaminations, as previously reported (Manfrevola et al., 2022). Following SCLB treatment, sperm cells were washed twice in PBS and revaluated under a light microscope to assess the efficiency of the treatment (data not shown). Lastly, an equal amounts of sperm cells were fixed by adding 875 µL of 1% formaldehyde and rocking at RT for 10 min. Cross-linking was quenched with 125 µL of 2 M glycine, rocked for 5 min at RT and centrifuged at 2000 × g for 5 min at 4°C. After quenching the sperm pellets were washed with PBS and stored at –80 °C for ChIP-seq experiments as following reported.

Sperm Lysis and Chromatin Immunoprecipitation (ChIP)

A pool of WT and $Cb1^{-/-}$ *caput* SPZ (n=5 for each experimental group) was used to perform ChIP-seq following previously published protocols (Yoshida et al., 2018) with some variations. Sperm pellets were washed twice with decondensation buffer (5 mM HEPES, pH 8.0, 0.2% NP-40, 10 mM EDTA, 5 mM NaCl, 1.2 M urea, supplemented freshly with 10 mM DTT, 1 mM PMSF, and protease inhibitors), then incubated in 1 mL of decondensation buffer supplemented with 1 mg/mL heparin and 10 mM DTT for 5 h at 42°C (or overnight at 37°C). Morphology was verified by light microscopy. Samples were washed twice with 1 mL of Wash Buffer 1 (50 mM HEPES, pH 7.5, 140 mM NaCl, 1 mM EDTA, 10% glycerol, 0.5% NP-40, 0.25% Triton X-100, freshly supplemented with 1 mM PMSF) for 5 min at RT. Pellets were resuspended in 200 µL of TE buffer (10 mM Tris-HCl, pH 8.0, 1 mM

EDTA) containing 1% SDS and protease inhibitors, and sonicated for 13 min (10 seconds on/10 seconds off cycles) on ice using a Misonix XL2020 sonicator at power setting 3. DNA fragmentation was verified on a 2% agarose gel to confirm a smear size between 200–500 bp. Sonicated chromatin was diluted with 1.8 mL of ChIP lysis buffer (10 mM Tris-HCl, pH 8.0, 100 mM NaCl, 1 mM EDTA, 0.5 mM EGTA, 1% Triton X-100, 0.1% sodium deoxycholate, supplemented with protease inhibitors). Samples were centrifuged at 16.000 × g for 10 min at 4°C, and the supernatant was collected. A 100 µL aliquot (5%) was reserved as input control (split equally for Western blotting and DNA control analyses). Protein A/G Dynabeads (Thermo Fisher) were washed with ChIP lysis buffer and incubated with 2–5 µg of histone H3 antibody or isotype-matched IgG for ≥2 h at 4°C under rotation. Antibody-coupled beads were then incubated with the chromatin lysate overnight at 4°C. After immunoprecipitation, the beads were washed four times with Wash Buffer 2 (50 mM HEPES, pH 7.0, 0.5 M LiCl, 1 mM EDTA, 0.7% sodium deoxycholate, 1% NP-40) and twice with TE buffer, each for 10 min at 4°C under rotation. Chromatin was eluted by adding 200 µL of TE buffer containing 0.2 mg/mL protamine sulfate and 1% SDS to the beads, and 130 µL of TE + 0.2 mg/mL protamine sulfate + 20 µL of 10% SDS to the input samples, followed by a 10-min incubation at RT with occasional mixing. Supernatants were collected for downstream applications (20 µL for Western blotting and 180 µL for DNA extraction).

Crosslink reversal was carried out by overnight incubation at 65°C. RNA was degraded by adding 5 µL of 10 mg/mL RNase A and incubating for 1 h at RT. Protein digestion was performed by adding 20 µL of 10 mg/mL Proteinase K and incubating at 60°C for at least 6 h. Subsequently, 16 µL of 5 M NaCl was added. The DNA was purified by two rounds of phenol:chloroform extraction followed by ethanol precipitation. Final cleanup was performed using the QIAquick PCR Purification Kit (Qiagen) according to the manufacturer's instructions. Purified DNA was used for downstream applications including qPCR and sequencing.

Sequencing

For ChIP-Seq experiments, samples were analyzed in service by the company Genomix4life using Illumina platform, that proceeded as here reported. Single-end sequencing was chosen, where short reads (75 base pair in length) are obtained from end of DNA fragments for ultra-high-throughput sequencing. Prior to further analysis a quality check was performed on sequencing data.

To perform this step, we used the tool for high throughput sequence data namely FastQC tool available on http://www.bioinformatics.babraham.ac.uk/projects/fastqc. The calculation of the Quality Value is performed based on the history "phred score”. Subsequently, the bioinformatic tool cutadapt (version 2.5) (Martin 2011) was used to remove the adapter sequence (if present) and the very short reads (reads length < 20). Then, the samples were mapped on reference mouse genome (MG39) (https://emea.support.illumina.com/sequencing/sequencing_software/igenome.html) using the bioinformatics tool using bowtie (Langmead et al., 2009), with the standard parameters. The alignment has been performed allowing 2 mismatches and retaining only uniquely mapped reads. The option --best --strata have been used also. The reference track was the assembly Mouse obtained from UCSC, by using iGenomes (https://emea.support.illumina.com/sequencing/sequencing_software/igenome.html). Instead, the redundant rate has been computed using Picard Tool (https://broadinstitute.github.io/picard/), MarkDuplicates module. The bioinformatics tool HOMER (http://homer.ucsd.edu/homer/index.html) (script findPeaks) was used to predict the binding sites. The following parameters were used:

i) -F (fold-enrichment over input tag): 2.0.

ii) -L (fold-enrichment over local tag count): 2.0.

iii) -C (fold-enrichment limit of expeted unique tag position): 1.0.

iv) -fdr: 0.01.

The peaks, in each sample have been annotated on reference mouse genome (https://emea.support.illumina.com/sequencing/sequencing_software/igenome.html). to compute how many are intergenic and how many are in promoter-, exonic- intronic-, 3' UTR- and 5'UTR- regions. The annotation has been performed using annotation script of HOMER software.
