# Supplementary material for: SIRT1 retention in elongating spermatids interferes with histone displacement by counteracting MOF-dependent H4K16 acetylation
Source: Front Cell Dev Biol. 2025 Aug 29;13:1524919. doi: 10.3389/fcell.2025.1524919 (PMC12426168; doi:10.3389/fcell.2025.1524919)
Supplement: Supplementary file 3 [file DataSheet3.pdf]

Supplementary Material

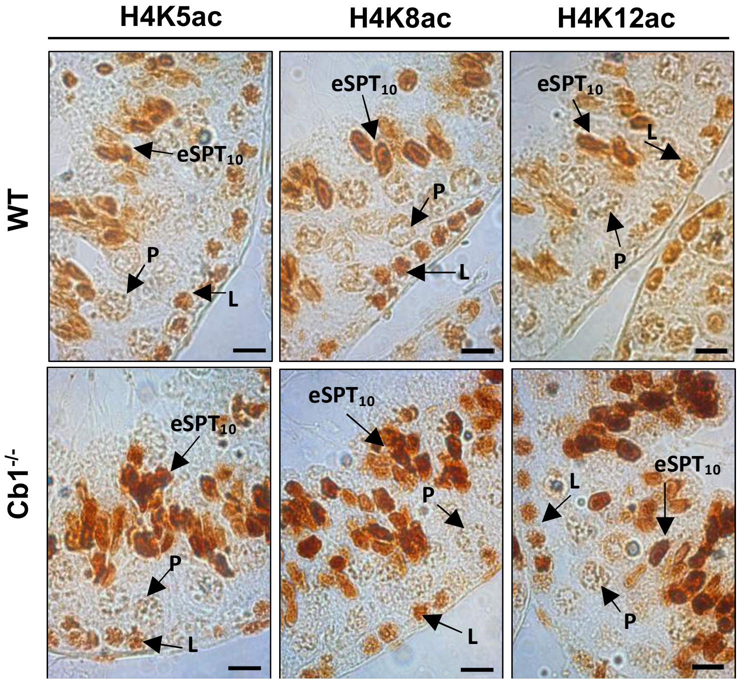

Figure S3. Immunohistochemistry analysis of H4K5ac, H4K8ac and H4K12ac in Bouin's fixed testicular sections (7 µm) of WT and Cb1$^{-/-}$ testes sections. The black arrowheads indicate the localization of H4K5ac, H4K8ac and H4K12ac in: spermatogonia (SPG), leptotene (L), pachytene (P) and elongating spermatids step 10 (eSPT$_{10}$). Scale bar: 20 µm.

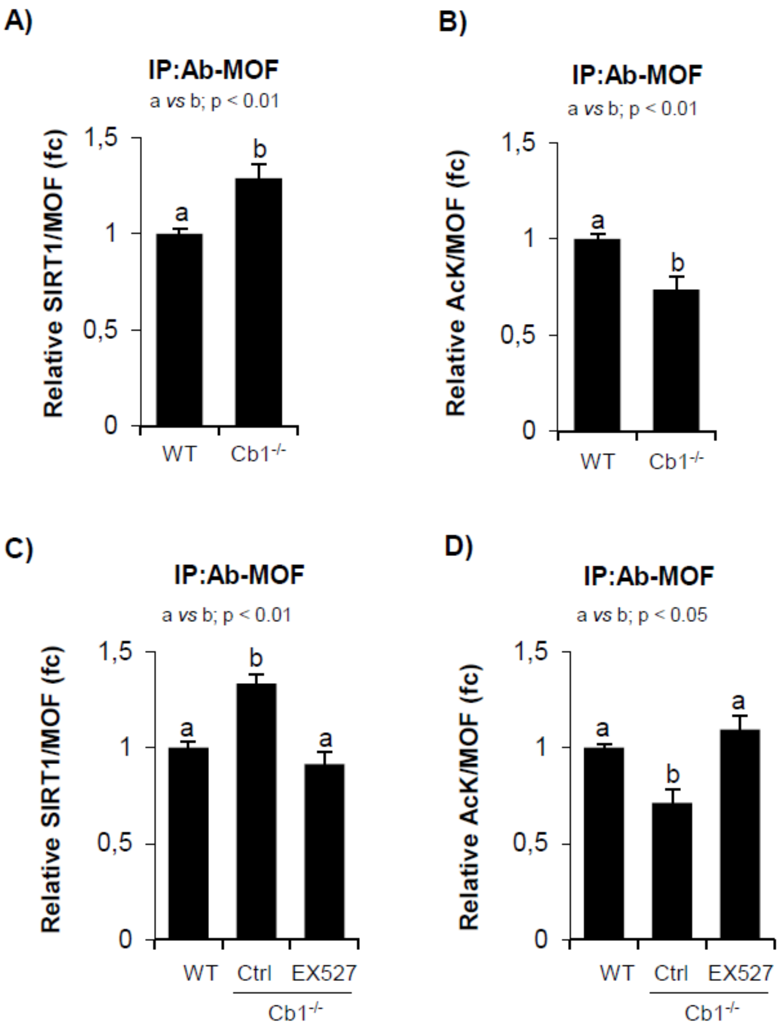

Figure S4. (**A-B**) Relative enrichment of SIRT1 (**A**) and AcK (**B**) in protein complexes immunoprecipitated using MOF antibody on total protein lysates from WT and $Cb1^{-/-}$ testis. (**C-D**) Relative enrichment of SIRT1 (**C**) and AcK (**D**) in protein complexes immunoprecipitated using MOF antibody on total protein lysates from WT and $Cb1^{-/-}$ testes *ex vivo* treated with vehicle (CTRL) or EX527. Protein amounts were quantified by densitometry analysis, normalized against the molecular bait MOF signal and expressed in OD values as fold change (fc). Data were reported as mean value ± S.E.M. Experimental groups with statistically significant differences were indicated with different letters.

Table S1: quantification of specific ROI withdrawn at germ cell nuclear level for immunostaining reported in figure 1.

	WT		Cb1 $^{+/-}$		Cb1 $^{-/-}$	
SIRT1	Mean SUM(I)/pixel^2	sd	Mean SUM(I)/pixel^2	sd	Mean SUM(I)/pixel^2	sd
SPG	91,716	9,278623	109,790	4,61424	127,907	11,73589
PL	92,021	3,523562	103,317	1,44038	127,458	10,98473
L	85,932	6,065562	108,573	2,35396	128,037	7,787367
Z	87,316	3,53412	89,123	3,51850	127,727	20,90632
P	103,129	0,642998	125,208	12,29179	137,840	10,07815
D	109,248	2,333877	130,365	3,161919	141,318	2,475581
$eSPT_8$	74,994	4,860383	112,817	0,630739	135,771	14,3974
$eSPT_9$	77,223	4,284595	101,981	0,868327	131,148	15,76353
$eSPT_{10}$	42,222	2,627739	56,166	7,944345	133,114	14,14143

Table S2: quantification of specific ROI withdrawn at germ cell nuclear level for immunostaining reported in figure 2.

	WT		Cb1 $^{+/-}$		Cb1 $^{-/-}$	
H4TtetraAc	Mean SUM(I)/pixel^2	sd	Mean SUM(I)/pixel^2	sd	Mean SUM(I)/pixel^2	sd
Stage VIII SPG	154,6522	2,2295	114,9243	11,2905	84,0608	6,1390
Stage VIII PL	156,5947	1,9174	103,7763	8,6437	98,6713	4,0020
Stage VIII P	93,0885	3,7124	84,4747	5,7603	59,7561	7,9755
Stage VIII SPT	168,7111	0,9910	131,7308	8,2284	89,0937	5,9049
Stage IX SPG	147,2319	2,4970	85,6723	11,9658	49,3440	7,5750
Stage IX L	73,0242	3,6804	95,2071	13,3562	49,5020	7,7142
Stage IX P	87,8757	5,7561	76,2785	4,6469	49,2607	8,4206
Stage IX SPT	180,6174	1,4298	139,2585	9,6029	107,7149	7,2361
Stage X SPG	118,4691	4,7945	60,6367	11,4255	48,1773	7,6975
Stage X L	98,9737	5,1448	91,0814	6,7903	49,0259	7,8015
Stage X P	91,8644	3,8171	56,6740	5,2336	50,0940	8,5235

Stage X SPT	178,3261	4,3082	177,8350	15,9960	127,1785	22,3913

Table S3: quantification of specific ROI withdrawn at germ cell nuclear level for immunostaining reported in figure 3.

	SPG		L		P		$eSPT_{10}$	
**WT**	Mean SUM(I)/pixel^2	sd	Mean SUM(I)/pixel^2	sd	Mean SUM(I)/pixel^2	sd	Mean SUM(I)/pixel^2	sd
SIRT1	90,996	1,35	96,256	1,00	105,324	3,22	63,624	6,38
H4K16ac	90,183	10,7	135,402	7,07	109,170	1,7	159,795	5,54
H4tetraAc	98,505	2,25	113,512	5,16	108,0855	3,11	159,606	1,05
**Cb1$^{+/-}$**	Mean SUM(I)/pixel^2	sd	Mean SUM(I)/pixel^2	sd	Mean SUM(I)/pixel^2	sd	Mean SUM(I)/pixel^2	sd
SIRT1	110,118	2,94	107,624	1,00	122,211	10,4	65,053	3,12
H4K16ac	68,446	8,52	104,906	5,64	76,428	6,87	159,587	4,3
H4tetraAc	65,309	1,51	92,085	2,01	66,123	2,96	158,663	7,91
**Cb1$^{-/-}$**	Mean SUM(I)/pixel^2	sd	Mean SUM(I)/pixel^2	sd	Mean SUM(I)/pixel^2	sd	Mean SUM(I)/pixel^2	sd
SIRT1	129,877	2,14	128,628	1,00	126,362	1,83	136,887	15,3
H4K16ac	74,559	6,33	98,629	2,79	73,423	3,85	110,505	7,93
H4tetraAc	63,423	2,9	71,713	6,91	64,328	2,85	101,991	6,47

Table S4: quantification of specific ROI withdrawn at germ cell nuclear level for immunostaining reported in supplemental figure 2.

	$eSPT_{10}$	
H3	Mean SUM(I)/pixel^2	sd
**WT**	115,035	35,31342
**Cb1$^{+/-}$**	151,671	55,5071
**Cb1$^{-/-}$**	196,737	9,374573

Table S5: quantification of specific ROI withdrawn at germ cell nuclear level for immunostaining reported in figure 4.

	SPG		L		P		$eSPT_{10}$	
**WT**	Mean SUM(I)/pixel^2	sd	Mean SUM(I)/pixel^2	sd	Mean SUM(I)/pixel^2	sd	Mean SUM(I)/pixel^2	sd
H4K16ac	103,555	10,13779	127,300	2,423962	105,675	0,991364	164,627	6,443864
H4tetraAc	106,493	9,878989	134,198	2,813966	111,519	3,063405	167,541	10,41093
**Cb1$^{+/-}$**	Mean SUM(I)/pixel^2	sd	Mean SUM(I)/pixel^2	sd	Mean SUM(I)/pixel^2	sd	Mean SUM(I)/pixel^2	sd
H4K16ac	71,113	15,70131	92,360	8,966114	87,072	2,114956	126,434	5,505533
H4tetraAc	70,520	8,78863	65,966	9,196917	62,502	5,406805	122,757	7,976674
**Cb1$^{-/-}$**	Mean SUM(I)/pixel^2	sd	Mean SUM(I)/pixel^2	sd	Mean SUM(I)/pixel^2	sd	Mean SUM(I)/pixel^2	sd
H4K16ac	68,488	5,096119	107,127	5,803932	69,874	2,170111	170,930	6,430429
H4tetraAc	65,393	7,078139	108,739	10,97412	71,160	3,865652	173,482	5,656806
