# Supplementary material for: SIRT1 retention in elongating spermatids interferes with histone displacement by counteracting MOF-dependent H4K16 acetylation
Source: Front Cell Dev Biol. 2025 Aug 29;13:1524919. doi: 10.3389/fcell.2025.1524919 (PMC12426168; doi:10.3389/fcell.2025.1524919)
Supplement: Supplementary file 4 [file DataSheet1.PDF]

Supplementary Material

Supplementary Data: Assessment of total H3 as histone displacement marker.

To confirm the reliability of total H3 in assessing histone displacement, we analysed H3 enrichment in step 10 eSPTs of WT, $Cb1^{+/-}$ and $Cb1^{-/-}$ mice, by IHC analysis. The content of VASA protein (also named DDX4), chosen as germ cell hallmark (Kim et al., 2015) was verified by western blot analysis to exclude interferences potentially depending on variations in germ cell content. As shown in figure S 2A, an enrichment of H3-labeled step 10 eSPTs was observed in $Cb1^{-/-}$ compared to WT and $Cb1^{+/-}$ mice. Consistently with our previous findings showing an increase in the observable SPZ with abnormal histone content in $Cb1^{-/-}$ mice (Cacciola et al., 2013), the IHC analysis revealed the presence of step 10 eSPTs lacking H3 labeling, indicative of efficient histone removal, in both WT and $Cb1^{+/-}$ mice but not observable in $Cb1^{-/-}$ ones. The VASA protein was equally expressed regardless of genotype (Fig. S 2B), confirming that the increase of total histone H3 observed in $Cb1^{-/-}$ testis was uniquely attributable to the defective histone displacement.

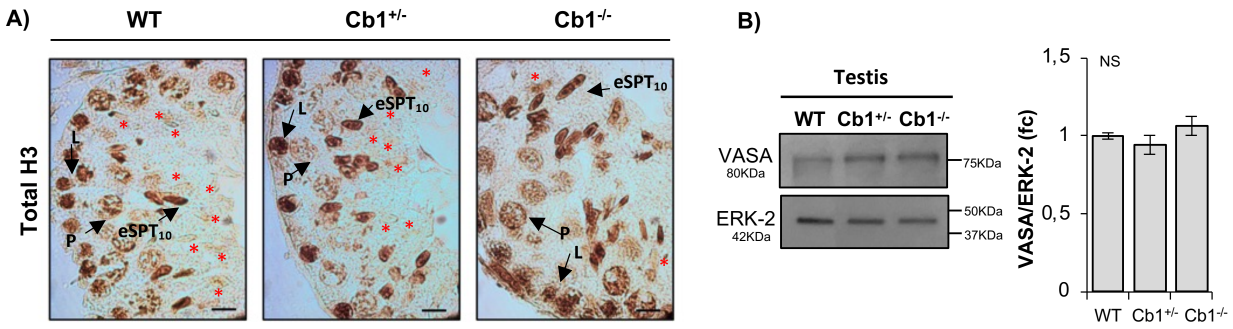

Figure S1. (**A**) Immunohistochemistry analysis of H3 in Bouin's fixed testicular sections (7 µm) of WT, $Cb1^{+/-}$ and $Cb1^{-/-}$ testes sections. The black arrowheads indicate the localization of H3 in: leptotene (L), pachytene (P) and elongating spermatids step 10 ($eSPT_{10}$). The red asterisks indicate $eSPT_{10}$ lacking H3 labeling. Scale bar: 20 µm. (**B**) Western blot analysis of VASA in WT, $Cb1^{+/-}$ and $Cb1^{-/-}$ testes. Protein amounts were quantified by densitometry analysis, normalized against ERK-2 signals and expressed in OD values as fold change (fc). Data were reported as mean value ± S.E.M.
